# Efficient Immunoglobulin Gene Disruption and Targeted Replacement in Rabbit Using Zinc Finger Nucleases

**DOI:** 10.1371/journal.pone.0021045

**Published:** 2011-06-13

**Authors:** Tatiana Flisikowska, Irmgard S. Thorey, Sonja Offner, Francesca Ros, Valeria Lifke, Bryan Zeitler, Oswald Rottmann, Anna Vincent, Lei Zhang, Shirin Jenkins, Helmut Niersbach, Alexander J. Kind, Philip D. Gregory, Angelika E. Schnieke, Josef Platzer

**Affiliations:** 1 Chair of Livestock Biotechnology, Technische Universität München, Freising, Germany; 2 Pharma Research and Early Development, Roche Diagnostics GmbH, Penzberg, Germany; 3 Sangamo BioSciences Inc., Richmond, California, United States of America; Brigham and Women's Hospital, United States of America

## Abstract

Rabbits are widely used in biomedical research, yet techniques for their precise genetic modification are lacking. We demonstrate that zinc finger nucleases (ZFNs) introduced into fertilized oocytes can inactivate a chosen gene by mutagenesis and also mediate precise homologous recombination with a DNA gene-targeting vector to achieve the first gene knockout and targeted sequence replacement in rabbits. Two ZFN pairs were designed that target the rabbit immunoglobulin M (IgM) locus within exons 1 and 2. ZFN mRNAs were microinjected into pronuclear stage fertilized oocytes. Founder animals carrying distinct mutated IgM alleles were identified and bred to produce offspring. Functional knockout of the immunoglobulin heavy chain locus was confirmed by serum IgM and IgG deficiency and lack of IgM^+^ and IgG^+^ B lymphocytes. We then tested whether ZFN expression would enable efficient targeted sequence replacement in rabbit oocytes. ZFN mRNA was co-injected with a linear DNA vector designed to replace exon 1 of the IgM locus with ∼1.9 kb of novel sequence. Double strand break induced targeted replacement occurred in up to 17% of embryos and in 18% of fetuses analyzed. Two major goals have been achieved. First, inactivation of the endogenous IgM locus, which is an essential step for the production of therapeutic human polyclonal antibodies in the rabbit. Second, establishing efficient targeted gene manipulation and homologous recombination in a refractory animal species. ZFN mediated genetic engineering in the rabbit and other mammals opens new avenues of experimentation in immunology and many other research fields.

## Introduction

Rabbits are important laboratory animals, widely used in many areas of biomedical research, including the production of antibodies and recombinant proteins. Rabbit models have contributed to the understanding of human diseases and the development of therapeutic compounds, devices and techniques. However it has not been possible to engineer precise genetic alterations in rabbits because they have so far been refractory to the two key enabling technologies; (I) rabbit embryonic stem (ES) cells capable of contributing to the germ line have yet to be derived, and (II) rabbits are particularly difficult to produce by somatic cell nuclear transfer [1].

The power and facility of gene targeting in ES cells has made the mouse by far the most intensively studied mammal [2]. Extending gene targeting to other species would deepen our understanding of gene function and further the development of many valuable biomedical applications, but the lack of fully functional ES cells has been a long-standing obstacle.

Nuclear transfer from cultured somatic cells (SCNT) was developed to circumvent the requirement for ES cells to generate gene-targeted animals. This is, however, technically difficult, and more than ten years since our first demonstration of targeting *COL1A1* in sheep [3], there are still few other examples: *PRNP* in sheep, cattle and goats [4]–[6], *GGTA1* in pigs [7], [8], *IGH* in cattle and pigs [5], [9], [10], *IGKC* in pigs [11] and *CFTR* in pigs [12].

Zinc-finger nucleases (ZFNs) are new tools that promise to radically simplify gene knockout and targeted gene replacement. An appropriately designed ZFN can create a double-strand break at a single predetermined site in the genomic DNA of an organism. In eukaryotes, double-strand break repair pathways often create small insertions and deletions at the break site, a useful means of inactivating genes of interest (for review, see Urnov et al. [13]). ZFN cleavage can also stimulate homology-directed genetic exchange between an episomal donor construct and a chromosomal locus, as first demonstrated for a native locus in Drosophila [14] and for endogenous loci in human cells [15]–[17].

A particularly promising approach is ZFN-mediated gene knockout directly in early embryos, because it offers a one-step method without any cell intermediate, as shown for zebrafish [18], [19], rats [20], [21] and mice [22]. Most recently, ZFN-mediated gene targeting by homologous recombination has been achieved in mice and rats [23], [24]. However ZFNs are likely to make their greatest impact in species where classical means of gene targeting are not available. Here we demonstrate that ZFNs enable precise genetic engineering in a particularly intractable species.

## Results

Given the failure of other techniques, we wished to investigate whether ZFN technology offers a practical means of targeted gene inactivation, addition or replacement in the rabbit. The immunoglobulin M locus was chosen as a suitable target because inactivation of endogenous immunoglobulins is a necessary first step for the production of human antibodies in a human immunoglobulin transgenic rabbit model.

### ZFN design and validation

**Figure 1:**
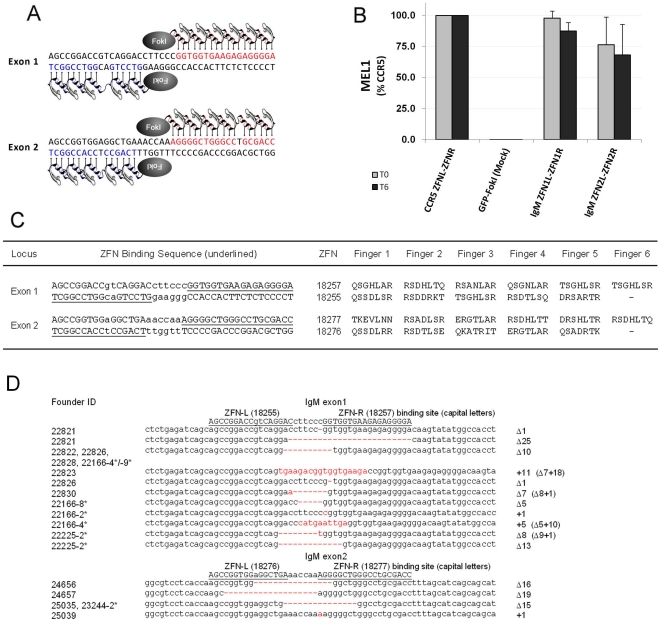
ZFN-mediated disruption of rabbit IgM. A. Recognition sequences in the rabbit IgM locus for ZFNs used in this study. B. Screening data using a budding yeast proxy system (see Materials and Methods) for ZFNs shown in panel A. The first sample represents positive control ZFNs that target the human CCR5 locus [51]. The grey and black bars represent reporter gene correction levels at low and high levels of ZFN synthesis, respectively. As detailed in Doyon et al. [18], the ZFN-encoding transgene is inducible, and this allows measurement of ZFN activity at different levels of the nuclease. C. DNA target sequences and recognition helices for the ZFNs used in the present work. The binding regions of left (ZFN-L) and right (ZFN-R) zinc finger protein array are underlined. Bases in capital letters are those that contribute to binding of the ZFP arrays. D. Summary of rabbit IgM mutant alleles obtained and characterized in offspring from ZFN microinjections. Stillborn animals are marked with an asterisk. The genotype of each allele is indicated to the right of the DNA sequence; inserted sequences are highlighted in red.

ZFNs directed against exons 1–4 of rabbit IgM (Figure S1) were designed using an archive of pre-validated zinc finger modules as described [14], [15], [17]–[20]. The ZFNs were ranked for activity using a budding yeast based system previously shown to identify nucleases active in editing endogenous loci in zebrafish and rat [18], [20], and the highest-ranking ZFN for each exon was selected for *in-vivo* use. Target sequence, structure and recognition helices of the selected ZFNs are shown in Figure 1 A–C.

### Establishment of rabbit oocyte microinjection conditions

The kinetics of ZFN activity in fertilized oocytes should be as rapid as possible, preferably before the first cleavage, to ensure that ZFN induced mutations are included in the germ line. ZFN activity should also be transient, to avoid possible accumulation of additional mutations in the developing embryo. In pilot experiments we microinjected EGFP mRNA diluted in either EDTA solution (0.1 mM, pH 8), or Tris/EDTA buffer (5 mM/0.1 mM, pH 7.5) into one of the two pronuclei or the cytoplasm of fertilized rabbit oocytes. Microinjection was verified in each oocyte by observing slight swelling of the pronucleus, or disturbance in the cytoplasm caused by solution streaming from the capillary. Of the oocytes injected into the pronucleus, only those injected with Tris/EDTA survived and developed further. Following cytoplasmic injection, survival and development was better in embryos injected with EDTA. Cytoplasmic microinjection of mRNA diluted in EDTA also led to earlier onset and higher EGFP expression at lower concentrations than pronuclear microinjection of mRNA in Tris/EDTA (Table S1).

We then examined technical parameters that could affect embryo viability and the levels of ZFN activity. These included ZFN mRNA concentration, site of microinjection (cytoplasm or pronucleus), and *in vitro* polyadenylation of mRNA. Injected embryos were cultured *in vitro* and monitored visually for development up to blastocyst stage. Exons 1 or 2 of the IgM locus were then amplified from blastocysts by nested PCR. Results indicated that: the optimal mRNA concentration range was between 3 to 6 ng/µl; microinjection of non-polyadenylated mRNA did not lead to effective mutagenesis; and microinjection into the pronucleus did not lead to effective mutagenesis (shown for exon 1 in Table S2). For gene inactivation experiments we therefore microinjected a 1∶1 mixture of polyadenylated mRNAs coding for the two components of a ZFN heterodimer, diluted to a total of 3 to 5 ng/µl in 0.1 mM EDTA, into the cytoplasm of pronuclear stage fertilized rabbit oocytes.

### Microinjection of ZFN mRNA produces rabbits mosaic at the IgM locus

mRNA coding for ZFN pair 18255/18257 targeted to IgM exon 1 (see Figure 1) was injected into 267 oocytes, 208 embryos were transferred into recipients and 17 live offspring were born. mRNA coding for ZFN pair 18276/18277 targeted to IgM exon 2 (see Figure 1) was injected into 259 oocytes, 212 embryos were transferred into recipients, and 17 live offspring were also born. Sequence analysis of the IgM locus in these 34 rabbits revealed that six animals carried mutations in exon 1, and four had mutations in exon 2. Eighteen stillborn rabbits were also analyzed. Mutations at the IgM locus were detected in six animals (Table 1), a similar proportion as observed in the live offspring. DNA sequence analysis of live and stillborn rabbits revealed a variety of mutations at the IgM locus, including deletions of 1 to 25 bp, single base insertions, and combinations of small deletions and insertions. Results are summarized in Figure 1D. Two live and one stillborn animal were found to carry two independent mutations at the IgM locus, suggesting that these were compound heterozygotes.

**Table 1:** Injection of ZFN-encoding mRNA into fertilized rabbit oocytes.

Target/ZFN Pair	Experiment	mRNA conc. (ng/**µ**l)	Oocytes injected/transferred	Oocytes per recipient	Offspring live/stillborn	F0 mutated (% of live offspring)	Mutations in F0	Founder (line) ID/gender	Mutated/total offspring in F1 (%)
IgM exon 118255/18257	1	3	40/22	11	2/1	1 (50)	Δ1; Δ25	22821/f	0/17° (0)
				11	1/0	1 (100)	Δ10	22822/f#	
	2	3	133/120	30	5/0	1 (20)	+11 (Δ7+18)	22823/m	1/80 (1.3)
				30	0/9	4* (0)	Δ5; Δ10; +1; +5 (Δ5+10)		
				30	7/2	3 (43)	Δ10; Δ1	22826/m	21**/83 (25)
							Δ10	22828/f§	
							Δ7 (Δ8+1)	22830/f	7/14 (50)
	3	5	94/66	22	1/1	0 (0)			
				22	1/2	1* (0)	Δ8; Δ13		
				22	0				
IgM exon218276/18277	4	3	96/75	20	1/0	1 (100)	Δ16	24656/f	2/5 (40)
				28	2/0	1 (50)	Δ19	24657/m	5/16 (31)
				27	0/2	1* (0)	Δ15		
	5	3	27/25	25	0/1	0 (0)			
	6	3	136/112	28	0/0	0 (0)			
				28	6/0	0 (0)			
				28	6/0	1 (17)	Δ15	25035/f	1/6 (17)
				28	2/0	1 (50)	+1	25039/m	2/13 (15)

*Stillborn.

° No further breeding.

# Exitus at 14 weeks of age.

§ Exitus at 19 weeks of age.

**10 offspring with 1 bp deletion; 11 offspring with 10 bp deletion.

### ZFN induced mutant IgM alleles are transmitted through the germline

Of the ten rabbits identified as carrying ZFN induced mutant IgM alleles, eight (four males and four females) survived to sexual maturity and were used as founders to produce a F1 generation. Seven F0 rabbits transmitted mutant IgM alleles at frequencies ranging from 1.3% to 50% (Table 1). Among these, one male (22826) and one female (22821) both carried two independent mutations. The female failed to pass on either mutation to the next generation, but the male transmitted both mutations at a similar rate, suggesting that founder rabbits had different degrees of mosaicism.

### IgM knockout rabbits are IgM and IgG deficient

In the immune response, the first antibodies to appear are of the IgM class. IgG, IgA, or IgE are then generated from IgM via class-switch recombination. Lack of IgM should therefore result in the absence of other isotypes. To determine if this was the case in our rabbits, we generated IgM knockout rabbits by breeding ZFN-treated founders and measured both IgM and IgG in serum.

**Figure 2:**
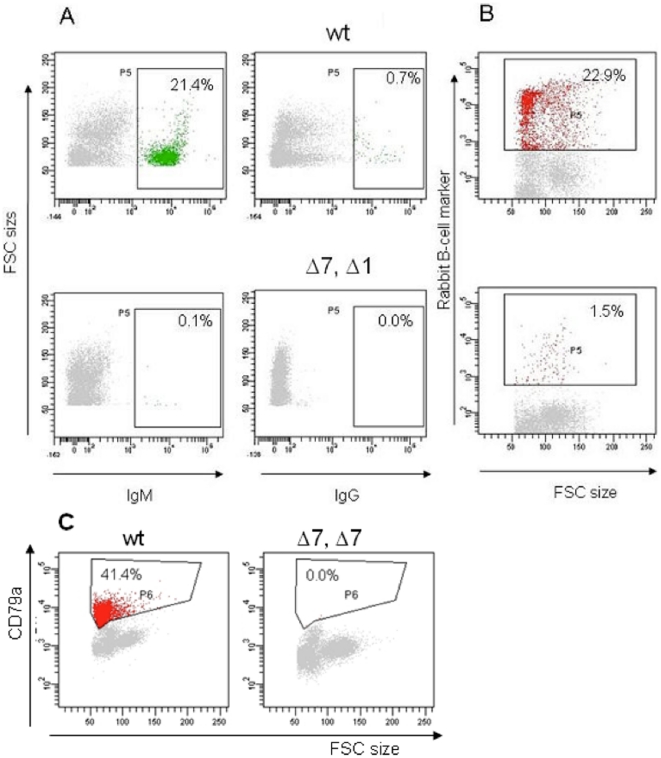
Characterization of the peripheral B-cell compartment of mutant offspring from ZFN-treated rabbits by FACS analysis. A. The top panels show representative dot plot profiles of peripheral blood mononuclear cells (PBMCs) of a wild type rabbit stained with antibodies specific for surface IgM (left) and surface IgG (right). Lower panels show Δ1/Δ7 PBMCs stained with the same antibodies. B. The upper dot plot shows wild type PBMCs immunostained with RACT30A, an antibody that recognizes a rabbit B-cell specific surface protein [25]. The lower panel shows Δ1/Δ7 PBMCs stained the same way. A and B: Only live PBMCs were included in these analyses. C. Dot plot profiles showing wild type (left) and Δ7/Δ7 (right) PBMCs, stained intracellularly for the B cell receptor component CD79a. The percentage of gated B cells (IgM^+^, IgG^+^, rabbit B cell marker^+^, or CD79a^+^) of all PBMCs is indicated in the upper right of each dot plot.

The Δ1 (male founder 22826) and Δ7 (female founder 22830) mutations in exon 1 both result in frameshifts in the IgM coding region and were predicted to disrupt expression of full-length protein. The founder male 22826 was intercrossed with the founder female 22830 to generate Δ1/Δ7 compound heterozygous animals. Three mutated F1 animals were obtained, one carrying the Δ1 mutation, one carrying the Δ7 mutation, and one Δ1/Δ7 compound heterozygote. The Δ7 mutation was also bred to homozygosity in an F2 generation. Serum IgM and IgG levels in these animals were determined by ELISA at 10 weeks of age. IgM and IgG levels were similar to wild type in Δ1 and Δ7 heterozygotes, but were undetectable in Δ1/Δ7 and Δ7/Δ7 rabbits (Table 2). The peripheral B-cell population in Δ1/Δ7 and Δ7/Δ7 rabbits was characterized by FACS analysis; only ∼0.1% of PBMCs stained positive for surface IgM (sIgM), and sIgG positive PBMCs were completely absent. In contrast, control wild type animals showed 21.4% of PBMC positive for sIgM and 0.7% positive for sIgG, typical for rabbit blood. A rabbit pan B-cell specific antibody [25] also revealed significantly fewer stained cells in the Δ1/Δ7 animal (1.5%) than in wild type (22.9%), see Figure 2 A and B. A second and well-defined B cell-specific marker is CD79a, constituting a part of the B cell receptor. Using an antibody that binds to the highly conserved intracellular domain of CD79a [26], no B cell receptor positive cells could be detected in Δ1/Δ7 and Δ7/Δ7 rabbits, whereas 41.4% of all PBMCs were marked as B cells in control animals (Figure 2 C).

**Table 2:** IgM and IgG serum levels at 10 weeks of age.

Parental lines	Genotype of offspring	Serum IgM (mg/ml)	Serum IgG (mg/ml)
22830×22826	Δ7, Δ1	bdl*	bdl*
22830×22826	Δ7, wt	2.7	1.4
22830×22826	Δ1, wt	1.1	1.3
25035×22826	Δ15, Δ1	1.5	0.9
22830×22830	Δ7, Δ7	bdl*	bdl*
Wild type (3 animals)	wt, wt	2.2	1.8
		1.7	5.7
		2.2	0.9

*Below detection limit: <500 ng/ml for rbIgM ELISA and <20 ng/ml for rbIgG ELISA.

The founder 22826 (Δ1 mutation) was also crossed with founder 25035, which carries a Δ15 deletion in exon 2 predicted to result in an in-frame loss of 5 amino acids. Serum analysis of Δ1/Δ15 F1 offspring revealed normal levels of IgM and IgG (Table 2). Thus, the Δ15 mutation does not appear to affect functional IgM expression or B-cell development.

These results show that ZFN-induced knockout alleles of the rabbit IgM gene transmit normally through the germline, and in homozygous form result in animals that are IgM- and IgG-deficient, but otherwise do not display obvious phenotypic abnormalities. It remains to be elucidated what effect the loss of IgM and IgG has on overall immunological function in these rabbits.

### ZFN-mediated targeted sequence replacement

**Figure 3:**
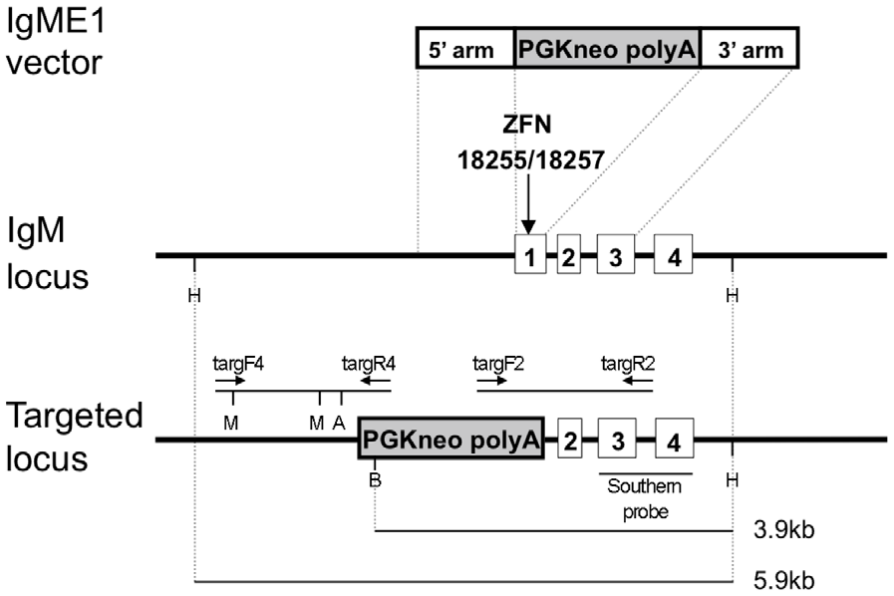
Targeted sequence replacement at the IgM locus. The structure of the IgME1 gene targeting vector, regions of homology with the IgM locus, and the predicted structure of the targeted locus are shown. Exons are marked and numbered as open boxes. The ZFN 18255/18257 cleavage site within exon 1 is indicated by a vertical arrow. The 5′ and 3′ junction PCR products are indicated; with positions of primers marked by arrows, and restriction sites for *MscI* and *ApaI* marked by (M) and (A), respectively. The hybridization probe and the diagnostic fragments used for Southern analysis are indicated in the lower part of the diagram. *HinDIII* (H) and *BspHI* (B) restriction sites are marked.

We then tested if ZFN-mediated targeted sequence replacement was feasible directly in the oocyte. A gene targeting vector, IgME1, was designed to replace IgM exon 1 with a PGK neo cassette, providing ∼1.9 kb of novel sequence. The strategy is illustrated in Figure 3; the full sequence of the gene-targeting vector is shown in Figure S2. Although the presence of the PGK neo gene was used for screening, no antibiotic selection was used to enrich for neo-positive embryos.

**Figure 4:**
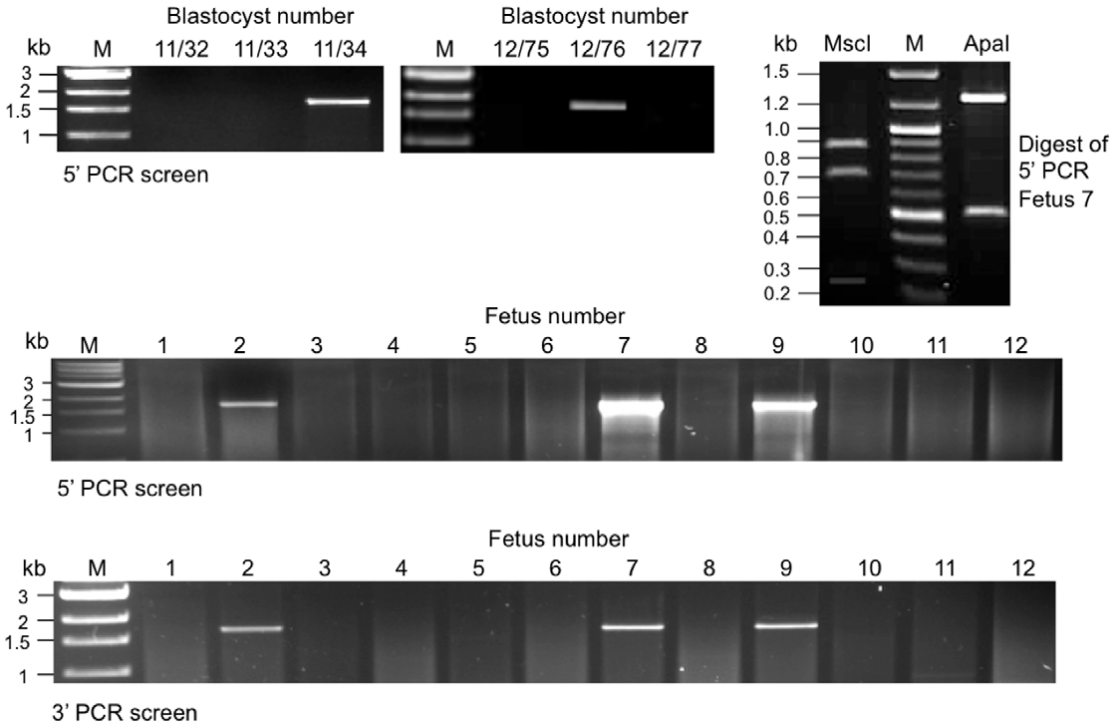
PCR analysis of gene targeted blastocysts and fetuses. Upper panel, representative 5′ junction PCR products amplified from blastocysts. Upper panel left, 5′ junction PCR from fetuses. Predicted 5′ PCR fragment size: 1.758 kb. Upper panel right, restriction digests of 5′ PCR fragment amplified from fetus 7. Predicted restriction fragment sizes: *ApaI* - 1242, 516 bp; *MscI* - 853, 687, 219 bp. Middle and bottom panels, 5′ and 3′ junction PCR products amplified from fetuses 1–12. Predicted 3′ PCR fragment size: 1.683 kb. M - size markers.

Mixtures of *MluI* linearised IgME1 DNA (concentrations ranging from 5 to 15 ng/µl) and ZFN 18255/18257 mRNAs (concentrations ranging from 3 to 9 ng/µl of each) were injected into pronuclear stage fertilized oocytes, see Table 3. Since ZFN mRNAs are most effective when injected into the cytoplasm, but IgME1 targeting vector DNA must be located in the nucleus to serve as the donor during homology-directed repair, we employed a two-step microinjection procedure similar to that described by Meyer et al. [23]. A portion of the mRNA/DNA mixture was first injected into a pronucleus to locate the vector DNA with the nuclear DNA. Then, on removing the injection needle, a second dose was injected into the cytoplasm to enable translation of ZFN mRNA. Microinjected oocytes were cultured to blastocyst stage and a total of 242 blastocysts were analyzed by PCR for targeted gene replacement events. Correct gene editing was detected in three blastocysts, as judged by the presence of a diagnostic 1.758 kb PCR product spanning the 5′ junction of the target site, see Figure 4. Data summarized in Table 3 show that concentrations of ZFN mRNA slightly higher than those used for mutagenesis were most effective in driving targeted sequence replacement. Combined data for all mRNA and targeting vector DNA concentrations indicated an overall rate of 1.2% targeted sequence replacement, but efficiency at the most effective mRNA/DNA concentrations was higher (17% at 9 ng/µl ZFN mRNA plus 10–15 ng/µl IgME1 DNA). Positive blastocysts were also screened for the presence of an intact IgM exon 1 by PCR. All gave a positive result, indicating that targeting had occurred at just one IgM allele.

**Table 3:** Screening of rabbit blastocysts for targeted gene replacement.

Concentration of ZFN mRNA (ng/**µ**l)	Concentration of vector DNA (ng/**µ**l)	Oocytes injected	Blastocysts screened	Blastocysts positive for targeting
3	5	102	45	0
3	10	39	26	0
6	5	76	43	0
6	10	118	76	1
6	15	28	18	0
9	10	17	6	1
9	15	60	28	1

Oocytes were then injected with 9 ng/µl ZFN 18255/18257 mRNA plus 10 ng/µl IgME1 DNA, and transferred to recipients. Seventeen fetuses were removed at day 15 of gestation and analyzed. All showed normal development, and examination of the uterine horns showed no evidence of degenerate or absorbed conceptuses. DNA was prepared from each fetus and screened by PCR. Three fetuses (2, 7 and 9) showed amplification of a 1.758 kb product across the 5′ junction and a 1.683 kb fragment across the 3′ junction of the target site, consistent with replacement of IgM exon 1 with the PGK neo cassette by homologous recombination, see Figure 4. The identity of the 5′ PCR product was confirmed by restriction digestion, see Figure 4. In addition, the DNA sequence of the 5′ PCR product from fetus 7 was determined and found to be identical to that predicted for the targeted locus, see Figure S2. Southern hybridization analysis was carried out using genomic DNA from the three PCR positive and five PCR negative fetuses. The Southern blotting scheme is outlined in Figure 3 and results shown in Figure S3. Each of the PCR positive fetuses showed a diagnostic restriction fragment spanning the 3′ junction of the target site, consistent with targeted replacement at the first exon, and also a fragment corresponding to a wild type IgM allele. The five negative fetuses showed only the wild type allele.

In summary, our data show efficient ZFN-induced gene disruption and homology-directed sequence replacement through microinjection in rabbit oocytes. We have obtained the first rabbits genetically engineered at a specific endogenous locus, the immunoglobulin M locus.

## Discussion

Gene targeting in ES cells has propelled the mouse to the forefront of biomedical research. This is despite the fact that its small size limits the scope for many physiological investigations and the low blood volume precludes its use as a source of serum factors. Metabolic and physiological differences also limit the relevance of mice to some human diseases, e.g. cardiomyopathy [27] or cystic fibrosis [12]. Other mammals, notably rabbit and pig, are increasingly seen as valuable alternatives but there is a significant need for efficient methods of precise genetic engineering in these species. Here we describe a generally applicable protocol for targeted genome editing in the rabbit. ZFNs introduced into fertilized oocytes can mediate gene inactivation by DNA repair mutagenesis and also homology-directed targeted sequence replacement. The technique is a simple, efficient and practical means of establishing lines of precisely genetically engineered rabbits.

Like mice, rabbits have a short generation time, produce large numbers of offspring and can be raised under specific pathogen free conditions. They have long been an important research animal, and are an indispensible source of polyclonal and monoclonal antibodies [28], [29]. Spontaneous mutant rabbit strains such as WHHL are widely used in the study of lipid metabolism and atherosclerosis [30]. Pronuclear microinjection of DNA is as facile in rabbits as in mice, and transgenic rabbits have been generated as models for retinal degeneration [31], cardiomyopathy [32], inflammation [33], hyperlipidemia [34] and for the evaluation of human vaccines [35]. Rabbits have also been used for the production of pharmaceutical proteins in milk. However, until now the scope of genetic manipulations has been limited to gain-of-function changes by classical random transgenesis.

Human polyclonal antisera produced by hyperimmunisation of animals with a humanized immune system are potentially an extremely useful new class of therapeutic agents. The inactivation of the IgM locus and loss of endogenous IgM and IgG expression on B cells and in serum that we describe is an essential step in the modification of rabbits to produce human immunoglobulins. The expression of immunoglobulin transgenes has been described for pigs, chicken, and rabbits [36]–[38], and recently the inactivation of immunoglobulin genes in pigs was reported [10], [11]. However, the inactivation of endogenous immunoglobulin genes in combination with addition of human immunoglobulin genes has so far only been achieved in mice and cattle [9], [39]–[44]. Experiments are now underway to achieve this in rabbits.

ZFN-mediated mutagenesis of the IgM locus in rabbit oocytes was efficient, yielding up to 30% mutant animals in the founder generation. Seven of eight founders passed on mutant alleles to their progeny. We also assessed whether ZFN-mediated targeted sequence replacement could be carried out directly in the oocyte without subsequent selection for the desired event, as recently described in mice [23]. Our results show targeted sequence replacement in rabbit at a rate of up to 18% in fetuses. The frequency of ZFN mediated homologous recombination events we observed was therefore at least equal to that described in mouse (1.7–4.5%, Meyer et al. [23]) and clearly higher than the rate of approximately 1 in 500 reported in mouse zygotes without ZFN [45]. These results suggest that the technique should be generally applicable, opening the way for the full repertoire of sophisticated gene replacement and modification techniques developed in mouse ES cells. ZFN-mediated genome editing does not require expression at the targeted locus and our data show that regions of homology as short as 1 kb or less, easily obtained by PCR amplification, are sufficient for efficient targeted sequence replacement. With less than 2 kb of total homology, our IgM gene targeting vector was considerably shorter than those described by Meyer et al. [23], but similar to those used for ZFN-mediated gene targeting in human transformed [16], ES and iPS cells [46].

Rodents, rabbits and other mammals all have particular limitations and strengths for biomedical research and are best regarded as complementary. Parallel and comparative studies performed in more than one species will speed progress in disease research and provide more reliable evaluation of novel diagnostic and therapeutic strategies. Gene targeting in ES cells is established in mouse and now in rat [3], but has so far been elusive in rabbit. Our approach circumvents the need for ES cells or SCNT to carry out precise alterations in any gene, because ZFNs can be designed against any native locus of interest [13], [47]. This achievement removes a major block to the development of genetically altered research models in those species where ES and SCNT technologies are not available. Furthermore, unlike ES cell-based methods, the desired genetic change can be introduced directly into the animal strain of choice, and heterozygous individuals obtained in the first generation. We are confident that this can be applied to other rabbit genes and other mammals, including important livestock species such as pigs. The possible applications are manifold.

## Materials and Methods

All animal experiments were approved by the Government of Upper Bavaria (permit numbers 55.2-1-54-2531-26-04 and 55.2-1-54-2531-9-09) and performed according to the European Union Normative for Care and Use of Experimental Animals. Chemical reagents were obtained from Sigma unless otherwise indicated.

### Gene targeting vector

The vector IgME1, used for ZFN-mediated targeted replacement, comprised a 0.758 kb homologous arm extending upstream of IgM exon 1 on the 5′ side, and a 1.035 kb arm extending downstream of IgM exon 1 to the end of exon 3 on the 3′ side, separated by a PGK neo polyA cassette (see Figure 3 and Figure S2). Homologous sequences were obtained by PCR from genomic DNA from a ZIKA rabbit.

### In-vitro mRNA transcription

Synthetic capped and poly A-tailed mRNAs were transcribed *in vitro* and purified using mMESSAGE mMACHINE T7 Ultra and MEGAclear Kits (Ambion). The quality and concentration of mRNAs were verified by denaturing gel analysis and spectrophotometry.

### Microinjection of ZFN mRNA and targeting vector DNA

Animals used as oocyte donors for the gene inactivation experiments were homozogous Alicia/homozogous Basilea back-crossed for several generations onto the ZIKA background. Alicia (Ali) rabbits carry a natural deletion within the IgH locus [48] and homozygous Basilea (Bas) animals do not express Igκ, due to a non-functional splice acceptor [49]. ZIKA rabbits were used as oocyte donors for the gene replacement experiments. Pronuclear stage fertilized oocytes were obtained by flushing oviducts 21 to 24 hrs from female rabbits superovulated by PMSG (Intergonan; Intervet) and hCG (Ovogest; Intervet) injection, inseminated artificially or by natural cover.

Microinjected oocytes were either cultured to blastocyst stage, or allowed to rest for 1 hr then transferred laparoscopically [50] to foster mothers primed by intramuscular injection of 0.2 ml Receptal (Intervet) 3 to 7 hrs previously. Pregnancies were either terminated at day 15 to examine fetuses, or allowed to proceed to term. Offspring were delivered normally and their genotype was determined at approximately 3 weeks of age.

### Detection of ZFN-induced gene inactivation and homologous recombination at the IgM locus

DNA was prepared from blastocysts by lysis in 50 mM KCl, 1.5 mM MgCl_2_, 10 mM Tris, pH 8.0, 0.5% NP40, 0.5% Tween20 and 100 µg proteinase K; or by lysis in 8 µl 50 mM NaOH (10 min 95°C; 2 µl 1 M Tris, pH 7.5 then added for neutralization); and from fetal tissue or ear biopsies of offspring by standard methods. Rabbit Cµ was amplified by PCR using primer pairs E1.1f (TGG CAG GGA CAC AGG AAA ACA) and E1.1r (GGC TCA CCG GGA AAG GAC); or E1.2f (GGC TGC CTG GCG CGG GAC TTT CT) and E1.2r (CTG TTG CTG TTG CTG TGC TGG ACT TTG) for exon1 and E2.1f (GGC TGC CTG GCG CGG GAC TTT C) and E2.1r (CCT GGC CTG GGG ACT GGA CAC TCA CT) or E2.2f (TGC CAG GCC ACA GGT TTC) and E2.2r (CTC GGA GGA CAT GGA CAC GTT CTT ATC) for exon 2. Nested PCRs from blastocysts were performed using the two primer pairs specific for exon 1 or 2 consecutively. PCR products containing mutated sequences detected by DNA sequence chromatography were cloned into the TOPO TA cloning kit (Invitrogen) for DNA sequencing of ZFN-induced mutations.

Targeted insertion of the PGK neo gene was detected by PCR across the 5′ junction using primers targF4 (GCT GTA CAG AGG TGA ACT GGG CTG GTC TTA) and targR4 (CCG CTG TAA GTC TGC AGA AAT TGA TGA TCT) and across the 3′ junction using primers targF2 (CGG AGA ACC TGC GTG CAA TCC ATC T) and targR2 (TGG GCA GAG AGA AGG TGG TGA GCC T). Southern hybridization of 10 µg samples of genomic DNA was performed with a digoxigenin dUTP labeled probe and detected by chemiluminescence using standard methods.

### Detection of rabbit IgM and IgG in serum

Serum antibody concentrations from animals between 7 and 15 weeks of age were determined by ELISA on streptavidin pre-coated 96 well microtiter plates (Micro Coat Biotechnologie, Germany). Capture was by anti-rabbit-IgM (μ-chain) or anti-rabbit-IgG (Fc) biotin (624085 and 550326, BD Pharmingen); detection used mouse anti-rabbit IgM or IgG horseradish peroxidase (BD Pharmingen).

### FACS analysis of peripheral blood mononuclear cells (PBMCs) of wild type and knockout rabbits

PBMCs were isolated from whole blood samples using separation media (Lympholyte-Mammal; Cedarlane) according to the manufacturer's instruction and stained and analysed immediately. For single colour flow cytometry, 5×10^5^ PBMCs were distributed as 50 µl aliquots in 1.5 ml tubes and kept on ice. Anti-rabbit IgG FITC was obtained from AbD Serotec (Düsseldorf, Germany). Anti-rabbit IgM biotin was from BD Pharmingen (San Diego, USA). FITC labelled streptavidin and anti-mouse IgM phycoerythrin (PE) conjugate were from Invitrogen (Germany). The monoclonal antibody RACT30A, which detects an unidentified rabbit B-cell surface marker [25], was obtained from VMRD (Pullman, USA). The PE-labelled antibody (clone HM47, BD Pharmingen), which binds the highly conserved intracellular domain of human CD79a (component of the B cell receptor) cross-reacts with rabbit CD79a [26]. For intracellular staining, PBMCs were fixed with Cytofix and permeabilised with Perm/Wash (BD Pharmingen) as mentioned in the instructions from the manufacturer. PBMC aliquots were incubated with 50 µl fluorochrome labelled mAbs or streptavidin in PBS for 30 min under rotation at 4°C in the dark. PBMCs were washed twice with ice cold PBS, resuspended in ice cold PBS and subjected to FACS analysis. For all surface staining, propidium iodide at a concentration of 5 µg/ml (BD Pharmingen, USA) was added prior to FACS analysis to discriminate between live and dead cells. 10,000 live PBMCs were analysed per sample. A FACSAria flow cytometer equipped with a computer and FACSDiva software (Becton Dickinson) was used to collect and analyse the data, FACS gates used are shown in each panel of Figure 2.

## Supporting Information

Figure S1 **IgM sequence polymorphisms in different rabbit strains.** The DNA sequence of the genomic IgM locus in 14 rabbits was determined; 6 rabbits of the ZIKA strain, 2 of the Alicia strain [48], which carry a mutation in the immunoglobulin heavy chain locus, and 6 of mixed breed (NZW/ZIKA/Alicia) carrying the Basilea [49] loss-of-function mutation at the immunoglobulin kappa light chain locus, which is on a different chromosome. Exon sequences are underlined (Exons: E1–E4). Polymorphisms are shaded in grey (R: A or G; Y: C or T). Five sequence polymorphisms were found in E1, two in E2, four in E3 and one in E4. The binding sites of the ZFNs used for microinjection are highlighted in red.
(TIF)

Figure S2 **DNA sequence of IgME-1 targeted locus.** The sequence of the IgME1 gene targeting vector is indicated by highlights; the 5′ and 3′ homologous arms in yellow and the PGK neo cassette in blue. The positions of primers used to amplify PCR fragments across the 5′ junction (targF4 and targR4) and across the 3′ junction (targF2 and targR2) are indicated. *ApaI* and *MscI* restriction sites used to confirm the identity of the 5′ PCR product (shown in Figure 3) and the *BspHI* site used for Southern analysis (Figure S3) are indicated.
(TIF)

Figure S3 **Southern analysis of fetuses.** Samples of genomic DNA from 9 of the 17 fetuses recovered (numbers indicated) were digested with *BspHI* and *HinDIII* and hybridized to a probe comprising IgM exon 3, intron 3 and exon 4. Arrows indicate positions of the diagnostic 5.911 kb *HindIII-HindIII* fragment, derived from the wild type allele, and the 3.943 kb *HindIII-BspHI* fragment, derived from the targeted allele.
(TIF)

Table S1 **Microinjection of mRNA coding for EGFP.** 1 Experiment.
(DOC)

Table S2 **Microinjection of mRNAs coding for ZFN SBS 18257/18255.** 4 Experiments (separated by double lines).
(DOC)
