# Supplementary figures and images for: Efficient Immunoglobulin Gene Disruption and Targeted Replacement in Rabbit Using Zinc Finger Nucleases
Source: PLoS One. 2011 Jun 13;6(6):e21045. doi: 10.1371/journal.pone.0021045 (PMC3113902; doi:10.1371/journal.pone.0021045)

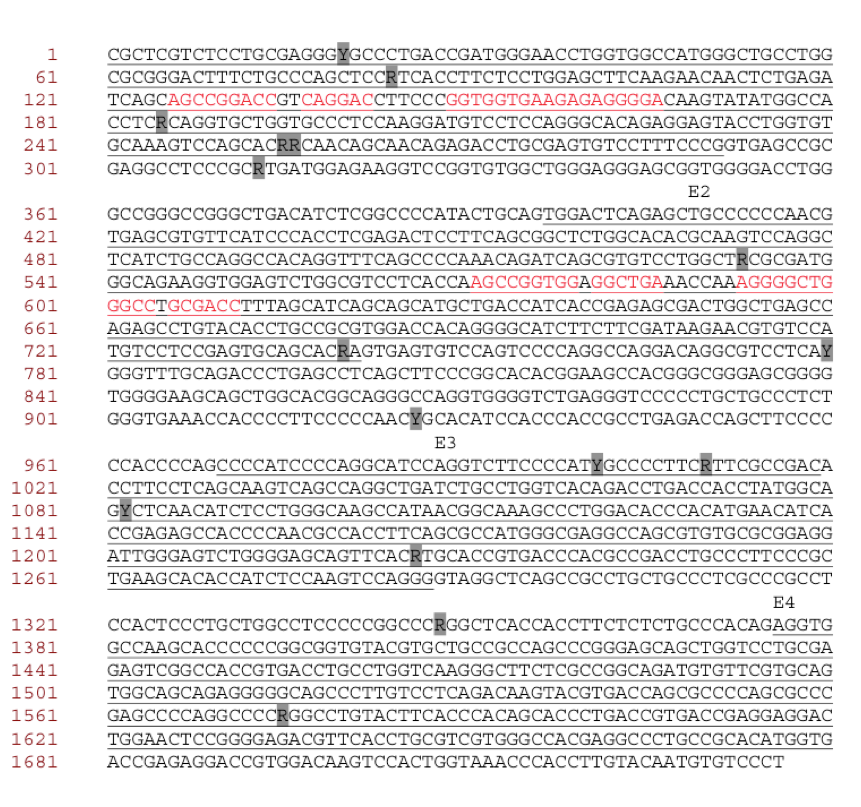

Supplement: Figure S1 — IgM sequence polymorphisms in different rabbit strains. The DNA sequence of the genomic IgM locus in 14 rabbits was determined; 6 rabbits of the ZIKA strain, 2 of the Alicia strain [48], which carry a mutation in the immunoglobulin heavy chain locus, and 6 of mixed breed (NZW/ZIKA/Alicia) carrying the Basilea [49] loss-of-function mutation at the immunoglobulin kappa light chain locus, which is on a different chromosome. Exon sequences are underlined (Exons: E1–E4). Polymorphisms are shaded in grey (R: A or G; Y: C or T). Five sequence polymorphisms were found in E1, two in E2, four in E3 and one in E4. The binding sites of the ZFNs used for microinjection are highlighted in red. (TIF) [file pone.0021045.s001.tif]

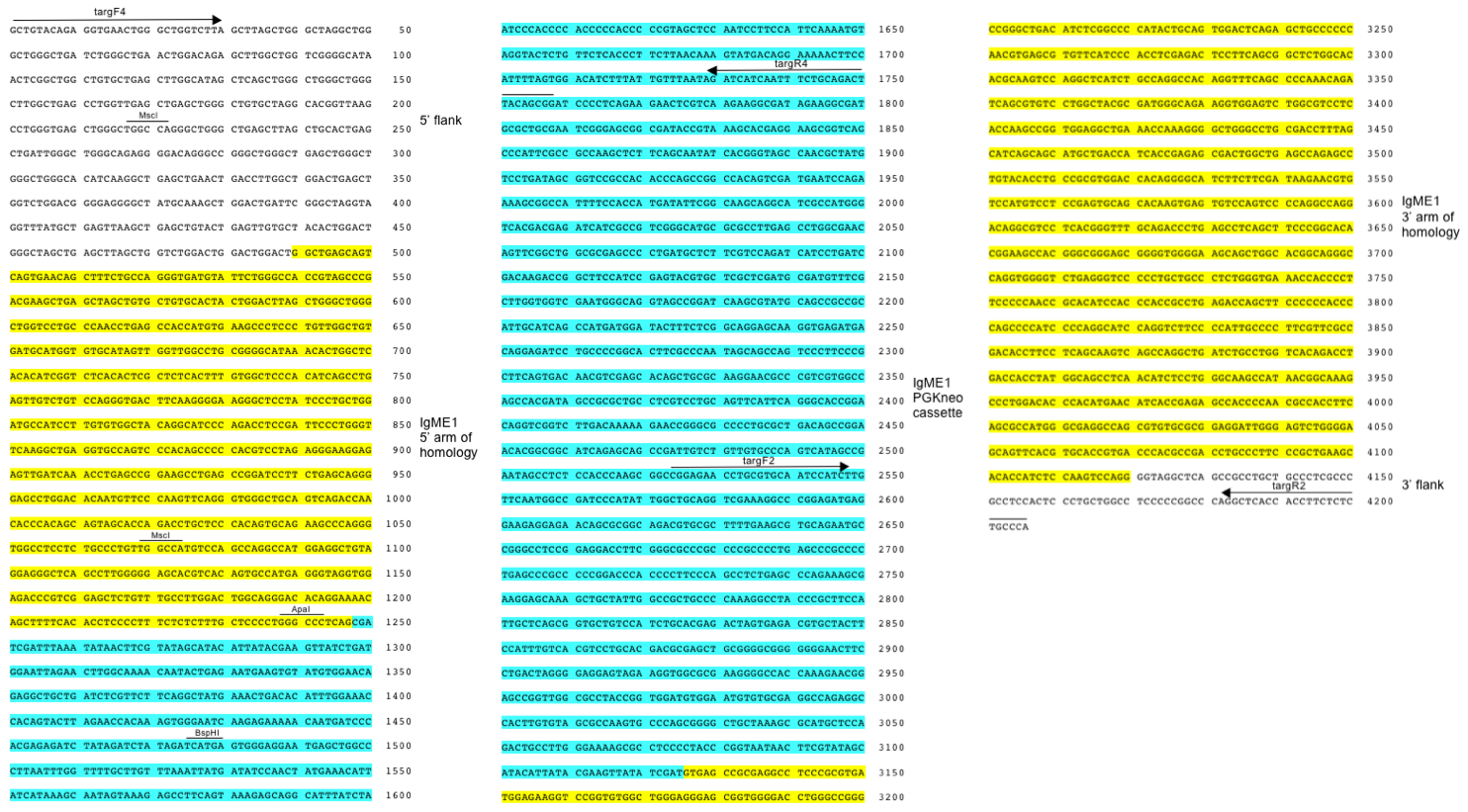

Supplement: Figure S2 — DNA sequence of IgME-1 targeted locus. The sequence of the IgME1 gene targeting vector is indicated by highlights; the 5′ and 3′ homologous arms in yellow and the PGK neo cassette in blue. The positions of primers used to amplify PCR fragments across the 5′ junction (targF4 and targR4) and across the 3′ junction (targF2 and targR2) are indicated. ApaI and MscI restriction sites used to confirm the identity of the 5′ PCR product (shown in Figure 3) and the BspHI site used for Southern analysis (Figure S3) are indicated. (TIF) [file pone.0021045.s002.tif]

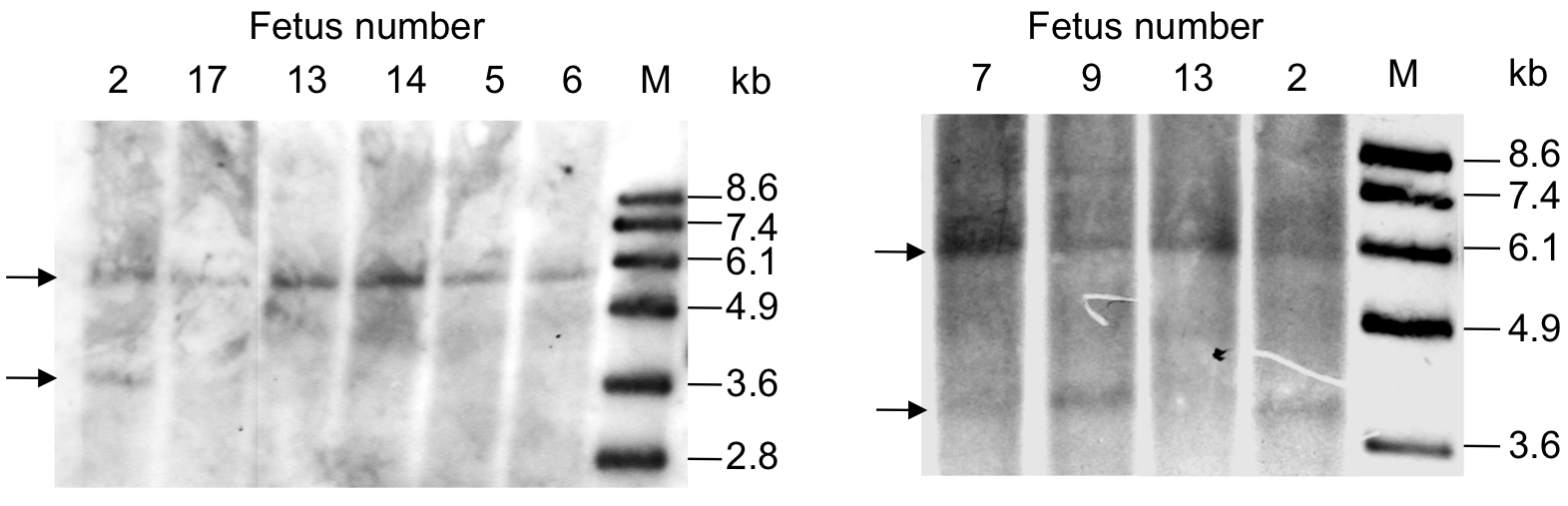

Supplement: Figure S3 — Southern analysis of fetuses. Samples of genomic DNA from 9 of the 17 fetuses recovered (numbers indicated) were digested with BspHI and HinDIII and hybridized to a probe comprising IgM exon 3, intron 3 and exon 4. Arrows indicate positions of the diagnostic 5.911 kb HindIII-HindIII fragment, derived from the wild type allele, and the 3.943 kb HindIII-BspHI fragment, derived from the targeted allele. (TIF) [file pone.0021045.s003.tif]
