# Supplementary material for: Efficient Immunoglobulin Gene Disruption and Targeted Replacement in Rabbit Using Zinc Finger Nucleases
Source: PLoS One. 2011 Jun 13;6(6):e21045. doi: 10.1371/journal.pone.0021045 (PMC3113902; doi:10.1371/journal.pone.0021045)
Supplement: Table S1 — Microinjection of mRNA coding for EGFP. 1 Experiment. (DOC) [file pone.0021045.s004.doc]

| **Injection route** | **Injection buffer** | **mRNA conc. (ng/µl)** | **Oocytes injected and cultivated** | **Development after 75 h*** | | | **EGFP expression** | | |
| --- | --- | --- | --- | --- | --- | --- | --- | --- | --- |
|  |  |  |  | **Cleavage**  **(%)** | **Morula**  **(%)** | **Blastocyst (%)** | **after 5 h**  **(%)** | **after 21 h**  **(%)** | **after 54 h**  **(%)** |
| Pronucleus | 0.1 mM EDTA /  5 mM Tris pH 7.5 | 10 | 9 | 9  (100) | 9  (100) | 6  (67) | 0 | 0* | 5 +  (56) |
|  |  | 50 | 11 | 10  (91) | 9  (82) | 5  (45) | 2 ++; 1 +  (27) | 10 +++  (91) | 7 +++; 3 ++  (91) |
|  | 0.1 mM EDTA | 10 | 10 | 0 | 0 | 0 | 1 +  (10) | 1 ++; 8 +  (90) | 1 +++; 4 +  (50) |
|  |  | 50 | 11 | 4  (36) | 0 | 0 | 4 +  (36) | 11 +  (100) | 8 +++ ^(m)^  (73) |
| Cytoplasm | 0.1 mM EDTA /  5 mM Tris pH 7.5 | 10 | 9 | 1  (11) | 7  (78) | 7  (78) | 0 | 0* | 7 +  (78) |
|  |  | 50 | 11 | 8  (73) | 8  (73) | 7  (64) | 4 ++; 3 +  (64) | 8 +++  (73) | 8 +++  (73) |
|  | 0.1 mM EDTA | 10 | 8 | 8  (100) | 6  (75) | 0 | 0** | 6 +  (75) | 4 ++; 4 +  (100) |
|  |  | 50 | 10 | 9  (90) | 9  (90) | 8  (80) | 3 ++; 3 +  (60) | 9 +++  (90) | 9 +++  (90) |

*, Normal rates of development for non-manipulated embryos from our previous experience are ~95 % development to morula and ~80 % development to blastocyst after 75 h; degeneration of the majority of embryos in an oocyte batch is observed occasionally

(m), Mosaic = expression in only a subset of cells in 4 embryos

**, Very weak expression at ~microscopic detection level
