# Supplementary material for: Efficient Immunoglobulin Gene Disruption and Targeted Replacement in Rabbit Using Zinc Finger Nucleases
Source: PLoS One. 2011 Jun 13;6(6):e21045. doi: 10.1371/journal.pone.0021045 (PMC3113902; doi:10.1371/journal.pone.0021045)
Supplement: Table S2 — Microinjection of mRNAs coding for ZFN SBS 18257/18255. 4 Experiments (separated by double lines). (DOC) [file pone.0021045.s005.doc]

| **Injection route** | **Injection buffer** | **In-vitro polyadenylation of mRNA** | **Concentration of mRNA (ng/µl)** | **Oocytes injected and cultivated** | **Development after 75 h*** | | | **PCR products obtained** | **Mutated sequences (%)** |
| --- | --- | --- | --- | --- | --- | --- | --- | --- | --- |
|  |  |  |  |  | **Cleavage**  **(%)** | **Morula**  **(%)** | **Blastocyst (%)** |  |  |
| Cytoplasm | 0.1 mM EDTA | + | 50 | 19 | 5  (26) | 1***  (5) | 0 | nd |  |
| Cytoplasm | 0.1 mM EDTA | + | 10 | 10 | 2  (20) | 2**  (20) | 1**  (10) | 2 | 2  (100) |
|  |  |  | 3 | 13 | 8  (62) | 8**  (62) | 4**  (31) | 5 | 4  (1 strong mosaic)  (80) |
| Cytoplasm | 0.1 mM EDTA | + | 6 | 33 | 22  (67) | 19**  (58) | 0** | nd |  |
|  |  | - |  | 37 | 26  (70) | 26  (70) | 26  (70) | nd |  |
| Pronucleus | 0.1 mM EDTA /  5 mM Tris pH 7.5 | + | 3 | 23 | 20  (87) | 20**  (87) | 14**  (61) | 21 | 0 |
|  |  | - |  | 23 | 18  (78) | 18  (78) | 14  (61) | 10 | 0 |
| Cytoplasm | 0.1 mM EDTA | + |  | 24 | 16  (67) | 16**  (67) | 6**  (25) | 16 | 8  (2 strong mosaic)  (50) |
|  |  | - |  | 24 | 10  (42) | 10  (42) | 7  (29) | 15 | 0 |

*, normal rates of development for non-manipulated embryos from our previous experience are ~95 % development to morula and ~80 % development to blastocyst after 75 h; degeneration of the majority of embryos in an oocyte batch is observed occasionally

**, morphology of embryos was normal, development was slightly retarded

***, morphology of embryo was abnormal
